# Target Approaching Control Under a GPS-Denied Environment with Range-Only Measurements

**DOI:** 10.3390/s25144497

**Published:** 2025-07-19

**Authors:** Bin Chen, Zhenghao Jing, Yinke Dou, Yan Chen, Liwei Kou

**Affiliations:** 1 College of Electrical and Power Engineering, Taiyuan University of Technology, Taiyuan 030024, China; 2023520511@link.tyut.edu.cn (B.C.); douyinke@tyut.edu.cn (Y.D.); chenyan@tyut.edu.cn (Y.C.); 2 Key Laboratory of Cleaner Intelligent Control on Coal & Electricity, Ministry of Education, Taiyuan University of Technology, Taiyuan 030024, China; 3 Shanxi Energy Internet Research Institute, Taiyuan 030032, China; 4 College of Mechanical Engineering, Taiyuan University of Technology, Taiyuan 030024, China; jingzhenghao7934@link.tyut.edu.cn

**Keywords:** localization, target approaching control, distance measurements

## Abstract

In this paper, we investigate the target-approaching control problem for a discrete-time first-order vehicle system where the target area is modeled as a static circular region. In the absence of absolute bearing or position information, we propose a simple local controller that relies solely on range measurements to the target obtained at two consecutive sampling instants. Specifically, if the measured distance decreases between two successive samples, the vehicle maintains a constant velocity; otherwise, it rotates its velocity vector by an angle of π/2 in the clockwise direction. This control strategy guarantees convergence to the target region, ensuring that the vehicle’s velocity direction remains unchanged in the best-case scenario and is adjusted at most three times in the worst case. The effectiveness of the proposed method is theoretically established and further validated through outdoor experiments with a mobile vehicle.

## 1. Introduction

The target approaching problem studied in this paper aims at designing a controller such that an autonomous vehicle can be steered to the vicinity of the target point. The practical motivation of this problem arises from applications to the autonomous landing of Unmanned Aerial Vehicles (UAVs). Specifically, vision-based methods have been intensively used for landing a UAV on a specified platform due to their advantages of strong autonomy, low cost, and strong anti-interference ability [1,2,3,4]. However, due to the short detectable distance and resolution of visual inspection, the autonomous landing of UAVs is implemented within only a limited distance. Thus, before vision is used for guidance, the UAV needs to be guided first to the vicinity of the landing area, which is in the line-of-sight range of the UAV.

Technically speaking, the target approaching problem is closely related to the target tracking problem, where a vehicle is required to capture a specified target [5,6,7], circumnavigate it [8,9,10], or pursue it from a distance. A common approach to addressing the target tracking problem involves the utilization of position information between the target and the vehicle. For example, by using the GPS information, a UAV calculates the relative position between the target and itself, such that the controller can be designed to drive the UAV to move towards the target. The target tracking control problem has been investigated via position-based information in the area of aircraft vehicles [11,12,13], mobile robots [14], and marine vehicles [15,16,17]. However, in the context of severe environments that render GPS signals jammed or spoofed, the vehicle is unable to acquire position information. In such scenarios, often referred to as a GPS-denied environment, vehicles fail to locate themselves and the target. Tremendous research efforts have been devoted to the target tracking mission by virtue of local measurements such as range and/or bearing under a GPS-denied environment. For instance, the problem of distributed target tracking for mobile sensor networks was investigated in [18], where the sensors use both range and bearing measurements of a target, and seek to minimize their distances to the target. An approximate tracking behavior was proposed in [19], where the range-and-bearing measurements are used to minimize the target’s location uncertainty. In [20], a target fencing controller was proposed using relative position information. It should be noted that relative position information in Cartesian coordinates is equivalent to the bearing and range information in polar coordinates.

In the aforementioned works, the results rely on the assumption that both the bearing (line-of-sight angle) and the range (relative distance between the vehicle and the target) are available for the control algorithm. There is also a relatively large body of research on target tracking with only bearing measurements, such as [21,22,23,24]. It is worth noting that the range-only method, also known as distance-based, is of particular value for the target tracking problem of vehicles not equipped with bearing sensors or in situations where the precise bearing measurement is unavailable. Over the past few decades, many efforts have been devoted to the target tracking problem with range-only measurements. In [25], a sliding mode control was proposed to steer a Dubins-like wheeled vehicle towards a target. The proposed strategy makes the vehicle rotate around the target with a predefined range margin from the target. In [26], the Equiangular Navigation Guidance (ENG) law was proposed for wheeled mobile robots to move towards an unknown stationary or maneuvering target. The authors in [27] proposed a switched logic-based control strategy to solve the pursuing problem for an autonomous robotic vehicle. In [28], the circumnavigation problem of a nonholonomic vehicle has been addressed by using range-only measurements. The range-based controller has also been considered in a few other relevant applications, such as source seeking [29,30,31], where the vehicle is required to seek the specified signal by measuring only the strength of the signal. The strength of the signal is assumed to decay away from the source through some physical processes.

In this paper, we are interested in the target approaching control problem when the only information available about the target is the range. In our particular case, the practical motivation arises from applications to the autonomous landing of Unmanned Aerial Vehicles (UAVs) in GPS-denied environments. In order to achieve the autonomous landing of UAVs, UAVs should be guided first to the vicinity of the landing area such that the vision-based method can be used to recognize the landing area. Note that neither the absolute position information nor the direction information to the landing area is available for the UAV. To overcome this problem, an alternative solution is for the UAV to deploy a range sensor, such as UWB, with which to measure the range from the landing area. As such, the range-based controller is utilized to guide the UAV to move to the vicinity of the landing area. The TAC problem in this paper aims at designing a local controller such that the vehicle is steered to the vicinity of the target of interest within a specified range. Not absolute bearing or position information is used for controller design. During the execution of the target approaching mission, the vehicle’s velocity is adjusted according to the range measurements from the target to itself at two consecutive sampling time instants. Specifically, the vehicle’s velocity remains constant if the range to the target decreases over two consecutive sampling time instants; otherwise, it undergoes a clockwise rotation by an angle of π/2 radians. It is proven that such a simple controller can solve the TAC problem in finite time instants. Optimization techniques are widely applied to address the target approach or target tracking problem, and many effective algorithms and theoretical results have been developed, such as model predictive control [32], control barrier function [33], machine learning-driven optimization [34], to name a few. These optimization-based methods often suffer from high real-time computation loads. In contrast with the above optimization-based methods, the proposed method can achieve the target approach task by only measuring the relative range between the target and the vehicle at two consecutive sampling times. Thus, the proposed method has superior temporal efficiency. The readers can refer to Remark 3 in this paper for the development compared with the current controller.

This paper is organized as follows. The problem formulation is given in Section 2. Section 3 presents the main results for the target approaching control problem. Simulation results are provided in Section 4 to validate the proposed method. Experimental results are presented in Section 5. Concluding remarks are given in Section 6.

Notation:Throughout this paper, we use the following notation: R denotes the field of real numbers and $Z^{+}$ is the set of positive integers. Given a vector $x\in R^{n}$, the Euclidean norm of *x* is denoted by ∥x∥. Given a real number y∈R, ⌈y⌉ denotes the ceiling function of *x*.

## 2. Problem Formulation

Consider a vehicle with the following discrete-time first-order form

(1) $$X_{k}=X_{k-1}+V_{k-1}T,$$

where $X_{k}\in R^{2}$ and $V_{k}\in R^{2}$ denote the position and velocity of the vehicle at the time kT, respectively, T>0 is the sample time interval, and $k\in Z^{+}$. The initial position and velocity are $X_{0}$ and $V_{0}$. It is worth noting that the vehicle maintains a constant velocity $V_{k}$ during the time interval ((k−1)T,kT]. The velocity $V_{k}$ is adjusted at the sample time kT by the proposed controller, which is introduced in the next section. As such, the vehicle will move along an uneven trajectory that consists of straight line segments. The target’s position is assumed to be a static point *O*. Define an area around the target as the following closed set

(2) Ω={X:∥X−O∥≤δ}.

Then, the main problem studied in this paper can be presented as follows.

The target approaching control (TAC) problem: Consider the vehicle dynamics (1) with the initial position $X_{0}\notin \Omega$ and the static target area (2). Design a control input $V_{k}$ such that the vehicle can enter the target area Ω in a finite step *k*.

It is noted that this paper focuses on the target approach control problem in the absence of environmental obstacles. Before presenting the formal theorems, some assumptions are given as follows.

**Assumption** **1.** *The norm of the control input $V_{k}$ remains a constant V before the vehicle enters the target area *Ω*, i.e., $∥V_{k}∥$ = V.*

**Remark** **1.** *It is noted that the core of the proposed TAC control law is to adjust the direction of the vehicle’s velocity to drive the vehicle to move toward the target while maintaining the magnitude of the vehicle’s velocity unchanged. It is a simple and effective strategy for the vehicle to reach the target area, especially in a complex environment. This idea derives from the extremum seeking to steer the vehicle to the target, where the controller is to keep the velocity constant and tune the velocity direction, a setting suitable for most autonomous vehicles [35,36].*

**Assumption** **2.** *At each sample time instant kT, the vehicle can measure the distance $d_{k}$ between the target and itself, i.e., $d_{k}=∥X_{k}-O∥$.*

**Remark** **2.** *In this paper, only the distance information $d_{k-1}$ and $d_{k}$ of two consecutive sample time instants is needed to design the control input $V_{k}$. No absolute orientation or position information is available for TAC.*

## 3. Main Results

In this section, the TAC problem is addressed using the following control strategy

(3) $$V_{k}=\left\{\begin{array}{cc} 0 & \;if\;d_{k}\leq \delta \\ V_{k-1} & \;if\;\delta <d_{k}\leq d_{k-1}\\ RV_{k-1} & \;if\;d_{k}>d_{k-1} \end{array}\right.,$$

where $d_{k}$ denotes the measured range between the vehicle and the target, and $R=[\begin{array}{cc} 0 & -1\\ 1 & 0 \end{array}]$ is a rotation matrix used to modify the velocity direction of the vehicle. By virtue of controller (3), the norm of the vehicle’s velocity is kept constant, and the direction of the vehicle’s velocity is adjusted such that the vehicle can be driven to the vicinity of the target.

**Figure 1:**
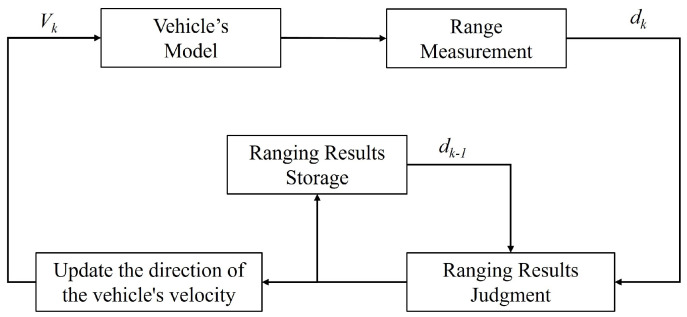
Block diagram of target approaching by keeping the magnitude of the vehicle’s forward velocity constant and adjusting the direction of its velocity. *d* is the distance between the vehicle and the target, $d_{k}$ is the current moment ranging result, and $d_{k-1}$ is the previous moment ranging result.

For clarifying the controller design, we consider the problem of target tracking using an autonomous vehicle. The vehicle’s task is to track a target of interest. We assume that the vehicle does not have the ability to sense its own position or the position of the target, but can obtain ranging data from the target. Keep the magnitude of the vehicle’s forward velocity constant and adjust the direction of its velocity through controller (3), which changes the vehicle’s direction to guide it toward the target region. This controller can be described by the block diagram in Figure 1. The distance *d* between the vehicle and the target is measured and transmitted to the TAC controller, which determines the trend of the distance measurements over consecutive time intervals. The current measurement result is stored for comparison with the subsequent measurement. If the range decreases, i.e., $d_{k-1}>d_{k}$, it indicates that the vehicle is approaching the target, and thus no adjustment to the direction of the vehicle’s velocity is required. Conversely, if the range increases, i.e., $d_{k-1}<d_{k}$, it implies that the vehicle is moving away from the target, and the controller will guide the vehicle to rotate $90^{\circ }$ clockwise to adjust its heading direction. By repeating this process, the vehicle can be steered to move toward the target location.

Now, we state the main result as follows.

**Theorem** **1.** *Consider a target area *(2)* and a vehicle in the form of *(1)*. Controller *(3)* solves the target approaching control problem under Assumptions 1 and 2 if the sample time interval T satisfies*

(4) $$T\leq \sqrt{\frac{2}{5}}\frac{\delta }{V}.$$

*In addition, the direction of the vehicle’s velocity is unchanged under the best-case scenario or is adjusted three times under the worst-case scenario during the TAC task.*

**Figure 2:**
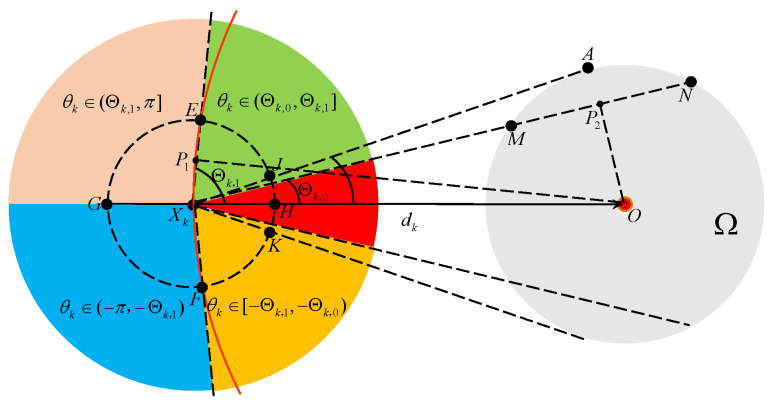
Illustration of the region division for $\theta_{k}$.

**Proof.**  For the convenience of proving Theorem 1, we introduce $\theta_{k}\in \left(-\pi ,\pi \right]$ to denote the clockwise angle from the vector $\vec{X_{k}X_{k+1}}$ to the vector $\vec{X_{k}O}$. Thus, $\theta_{k}$ represents the direction error between the ideal moving direction $\vec{X_{k}O}$ and the real moving direction of the vehicle at the kT time instant. From a practical perspective, the direction error $\theta_{k}$ is critical for analyzing the TAC problem. As shown in Figure 2, the whole region is divided into five parts for $\theta_{k}$. Specifically, draw a line $X_{k}A$ tangent to the boundary of the target area at the point *A*, and draw a secant line $X_{k}N$ intersecting the boundary of the target area at two points *M* and *N* such that ∥MN∥=VT. In addition, draw a circle with radius VT and center $X_{k}$, and another circle with radius $d_{k}$ and center *O*. These two circles intersect at two points *E* and *F*. Let $P_{1}$ and $P_{2}$ be the orthogonal projections of *O* onto $X_{k}E$ and MN, respectively. A straightforward geometric analysis yields $∥X_{k}P_{1}∥\;=VT/2$ and $∥OP_{2}∥\;=\sqrt{\delta^{2}-{\left(VT\right)}^{2}/4}$. Then, two angle thresholds $\Theta_{k,0}$ and $\Theta_{k,1}$ are defined by

(5) $$\begin{array}{cc} \Theta_{k,0} & =\arcsin\frac{∥OP_{2}∥}{d_{k}}=\arcsin\frac{\sqrt{\delta^{2}-{\left(VT\right)}^{2}/4}}{d_{k}}, \end{array}$$

(6) $$\begin{array}{cc} \Theta_{k,1} & =\arccos\frac{∥X_{k}P_{1}∥}{d_{k}}=\arccos\frac{VT}{2d_{k}}, \end{array}$$

where $0<\Theta_{k,0}<\Theta_{k,1}$ due to $d_{k}>\delta$. $\Theta_{k,0}$ and $\Theta_{k,1}$ play a key role during the TAC process in the following manner.

- $|\theta_{k}|\;\in \left(\Theta_{k,1},\pi \right]$. In this case, $X_{k+1}\in \overset{⌢}{EGF}$, $d_{k+1}>d_{k}$ hold and $V_{k+1}=RV_{k}$ according to controller (3).
- $|\theta_{k}|\;\in \left[0,\Theta_{k,1}\right]$. In this case, $X_{k+1}\in \overset{⌢}{EHF}$, $d_{k+1}\leq d_{k}$ holds and $V_{k+1}=V_{k}$ according to controller (3). In particular, if $|\theta_{k}|\;\in \left[0,\Theta_{k,0}\right]$ holds, there exist two intersection points between the ray $X_{k}X_{k+1}$ and the boundary of Ω.

According to the division of the direction error $\theta_{k}$, Theorem 1 is proven in five cases. For ease of expression, define ϑ(k) by $\vartheta \left(k\right)=\arcsin\left(\frac{VT-d_{k-1}\cos\theta_{k-1}}{d_{k}}\right)$. If $\delta \geq \sqrt{\frac{5}{2}}VT$ holds, we have that

(7) $$\arcsin\left(\frac{3VT}{2d_{k}}\right)\leq \Theta_{k,0}$$

in light of (5). The result is vital and will be used to prove that the direction error $|\theta_{k}|\;\in \left[0,\Theta_{k,0}\right]$ is satisfied after the vehicle’s velocity is rotated a few times. According to the value of $\theta_{k}$, the Theorem 1 is proved in five cases. □

### 3.1. In the Case of $|\theta_{k}|\;\in \left[0,\Theta_{k,0}\right]$

**Figure 3:**
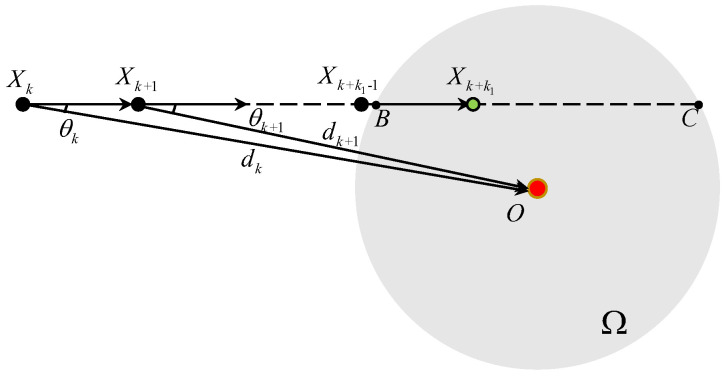
The TAC process for $\theta_{k}\in \left(\Theta_{k,0},\Theta_{k,1}\right]$.

When $|\theta_{k}|\;\in \left[0,\Theta_{k,0}\right]$, there are two intersection points *B* and *C* between the ray $X_{k}X_{k+1}$ and the boundary of Ω as displayed in Figure 3. A simple calculation gives $∥BC∥=2\sqrt{\delta^{2}-{\left(d_{k}\sin\theta_{k}\right)}^{2}}\geq 2\sqrt{\delta^{2}-{\left(d_{k}\sin\Theta_{k,0}\right)}^{2}}=VT$. It indicates that there exists at least a $K\in Z^{+}$ such that $X_{K}\in \Omega$ due to the fact that the $∥X_{i}-X_{i-1}∥\;=VT$. In this case, $d_{i}<d_{i-1}$ always holds before the vehicle enters Ω. Thus $V_{i}=V_{k}$ always holds for $i\in \{k+1,\cdots ,k+k_{1}-1\}$, where

(8) $$k_{1}=\left\lceil \frac{∥X_{k}B∥}{VT}\right\rceil .$$

At the $(k+k_{1}-1)T$ time instant, the position of the vehicle satisfies $∥X_{k}X_{k+k_{1}-1}∥=\left(k_{1}-1\right)VT<∥X_{k}B∥$. At the $(k+k_{1})T$ time instant, we have $∥X_{k}X_{k+k_{1}}∥\geq ∥X_{k}B∥$ and $∥X_{k}X_{k+k_{1}}∥<∥X_{k}B∥+VT=\;∥X_{k}C∥$. Thus, $∥X_{k}X_{k+k_{1}}∥\;\in \;[∥X_{k}B∥,∥X_{k}C∥)$ and $X_{k+k_{1}}\in \Omega$ holds. The TAC problem is solved with the vehicle’s velocity unchanged.

Thus, under Assumptions 1 and 2, if $|\theta_{k}|\;\in \left[0,\Theta_{k,0}\right]$, the TAC problem is solved in a finite step *k* by controller (3) with the vehicle’s velocity unchanged.

The above results indicate that if the vehicle moves toward the target with the initial angle $|\theta_{k}|\;\in \left[0,\Theta_{k,0}\right]$, it can enter the target area without changing the direction of its velocity. For the case of $|\theta_{k}|\;\notin \left[0,\Theta_{k,0}\right]$, it is proved in what follows that the vehicle will adjust its velocity by controller (3) such that at the $k^{\prime }T$ time instant, $|\theta_{k^{\prime }}|\;\in \left[0,\Theta_{k^{\prime },0}\right]$ holds with $k^{\prime }\in Z^{+}$.

### 3.2. In the Case of $\theta_{k}\in \left(\Theta_{k,0},\Theta_{k,1}\right]$

It is observed from Figure 2 that if $\theta_{k}\in \left(\Theta_{k,0},\Theta_{k,1}\right]$, the number *N* of the intersection points between the ray $X_{k}X_{k+1}$ and the boundary of Ω satisfies that N∈{0,1,2}.

#### 3.2.1. N=2

**Figure 4:**
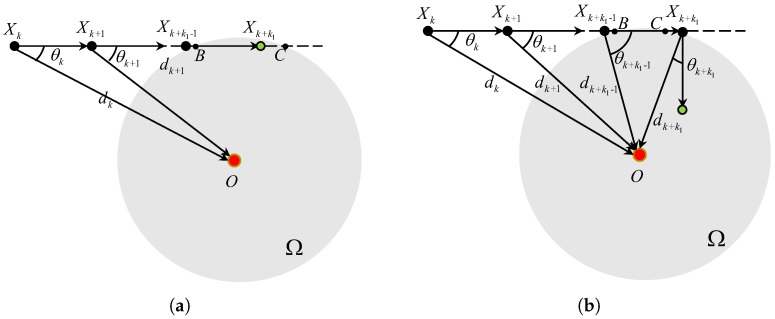
The TAC process for $\theta_{k}\in \left(\Theta_{k,0},\Theta_{k,1}\right]$ and N=2. (**a**) Equation (9) holds. (**b**) Equation (10) dose not holds.

As shown in Figure 4, if N=2, the two intersection points *B* and *C* between the ray $X_{k}X_{k+1}$ and the boundedness of Ω satisfy that $0<∥BC∥=2\sqrt{\delta^{2}-{\left(d_{k}\sin\theta_{k}\right)}^{2}}<2\sqrt{\delta^{2}-{\left(d_{k}\sin\Theta_{k,0}\right)}^{2}}=VT$. At the $(k+k_{1}-1)T$ time instant where $k_{1}$ is defined by Equation (8), we have that $∥X_{k}X_{k+k_{1}-1}∥=\left(k_{1}-1\right)VT<∥X_{k}B∥$ and $∥X_{k}X_{k+k_{1}}∥\geq ∥X_{k}B∥$. Whether the TAC problem can be solved at the $(k+k_{1})T$ time instant depends on $∥X_{k}X_{k+k_{1}}∥$ and $∥X_{k}C∥$. Specifically, if

(9) $$\left\lceil \frac{∥X_{k}B∥}{VT}\right\rceil \leq \frac{∥X_{k}C∥}{VT}$$

holds, we have that $∥X_{k}X_{k+k_{1}}∥=k_{1}VT\leq ∥X_{k}C∥$. It, together with the fact that $∥X_{k}X_{k+k_{1}}∥\;\geq \;∥X_{k}B∥$ indicates that $∥X_{k}X_{k+k_{1}}∥\;\in [∥X_{k}B∥,∥X_{k}C∥)$ and $X_{k+k_{1}}\in \Omega$ hold.

Conversely, if Equation (9) does not hold, we have that $∥X_{k}X_{k+k_{1}}∥>∥X_{k}C∥$ and $∥X_{k}X_{k+k_{1}-1}∥<∥X_{k}B∥$. It implies that the TAC problem is not solved at the $(k+k_{1})T$ time instant, i.e., $X_{k+k_{1}}\notin \Omega$. Regarding this case, if $d_{k+k_{1}}>d_{k+k_{1}-1}$, we have $V_{k+k_{1}}=RV_{k+k_{1}-1}$ and $\theta_{k+k_{1}-1}>\Theta_{k+k_{1}-1,1}$. The angle error $\left|\theta_{k+k_{1}}\right|=\left|\vartheta \left(k+k_{1}\right)\right|\leq \arcsin\left(\frac{3VT}{2d_{k+k_{1}}}\right)$. According to Equation (7), we have that $\left|\theta_{k+k_{1}}\right|\leq \Theta_{k+k_{1},0}$, as displayed in Figure 4b. If $d_{k+k_{1}}\leq d_{k+k_{1}-1}$, we have $V_{k+k_{1}}=V_{k+k_{1}-1}$ and $\theta_{k+k_{1}}>\pi /2>\Theta_{k+k_{1},1}$. Thus, $d_{k+k_{1}+1}>d_{k+k_{1}}$, $V_{k+k_{1}+1}=RV_{k+k_{1}}$, and the angle error $\left|\theta_{k+k_{1}+1}\right|=\left|\vartheta \left(k+k_{1}+1\right)\right|\leq \arcsin\left(\frac{3VT}{2d_{k+k_{1}+1}}\right)\leq \Theta_{k+k_{1}+1,0}$.

#### 3.2.2. N=1

**Figure 5:**
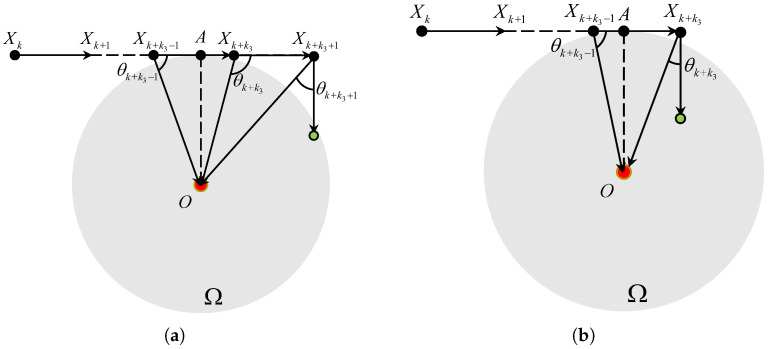
The TAC process for $\theta_{k}\in \left(\Theta_{k,0},\Theta_{k,1}\right]$ and N=1. (**a**) $k_{3}-k_{2}\leq 0.5$. (**b**) $k_{3}-k_{2}>0.5$.

In this case, the ray $X_{k}X_{k+1}$ is tangent to the boundary of Ω at the point *A* as plotted in Figure 5. Then, it is easily observed that the TAC problem is solved at the point *A* if

(10) $$k_{2}=\frac{∥X_{k}A∥}{VT}\in Z^{+}.$$

Conversely, if $k_{2}\notin Z^{+}$, we define

(11) $$k_{3}=\left\lceil \frac{∥X_{k}A∥}{VT}\right\rceil .$$

As shown in Figure 5a, if $k_{3}-k_{2}\leq 0.5$, we have $\theta_{k+k_{3}-1}\leq \Theta_{k+k_{3}-1,1}$ and $\theta_{k+k_{3}}>\Theta_{k+k_{3}-1,1}$. It means that $d_{k+k_{3}}\leq d_{k+k_{3}-1}$, $V_{k+k_{3}}=RV_{k+k_{3}-1}$, $d_{k+k_{3}+1}>d_{k+k_{3}}$ and $V_{k+k_{3}+1}=RV_{k+k_{3}}$. The angle error $\left|\theta_{k+k_{3}+1}\right|=\left|\vartheta \left(k+k_{3}+1\right)\right|\leq \arcsin\left(\frac{3VT}{2d_{k+k_{3}+1}}\right)\leq \Theta_{k+k_{3}+1,0}$. As shown in Figure 5b, if $k_{3}-k_{2}>0.5$, we have $\theta_{k+k_{3}-1}>\Theta_{k+k_{3}-1,1}$, $d_{k+k_{3}}>d_{k+k_{3}-1}$ and $V_{k+k_{3}}=RV_{k+k_{3}-1}$. The angle error $\left|\theta_{k+k_{3}}\right|=\left|\vartheta \left(k+k_{3}\right)\right|\leq \arcsin\left(\frac{3VT}{2d_{k+k_{3}}}\right)\leq \Theta_{k+k_{3},0}$.

#### 3.2.3. N=0

**Figure 6:**
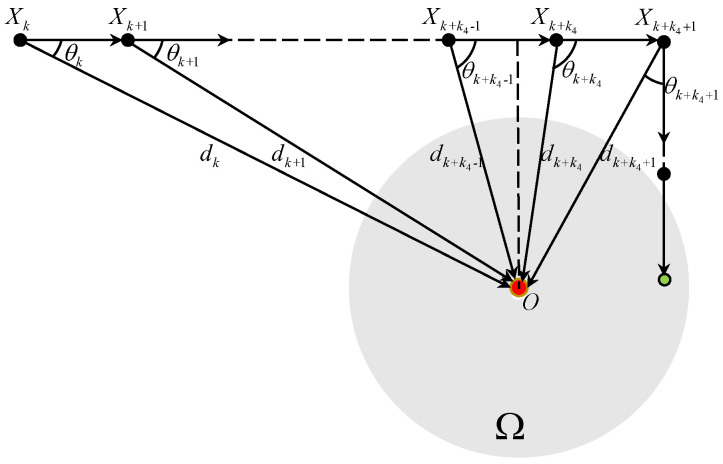
The TAC process for $\theta_{k}\in \left(\Theta_{k,0},\Theta_{k,1}\right]$ and N=0.

Figure 6 plots the TAC process for N=0. If $\theta_{k}\in \left(\Theta_{k,0},\Theta_{k,1}\right]$, we have $d_{k+1}\leq d_{k}$ and $V_{k+1}=V_{k}$. The angle error $\theta_{k+1}$ satisfies that $\sin\theta_{k+1}=\frac{d_{k}}{d_{k+1}}\sin\theta_{k}\geq \sin\theta_{k}$. Let a finite $(k+k_{4})T$ be the first time when $\theta_{k+k_{4}}>\Theta_{k+k_{4},1}$ holds, we have $d_{k+k_{4}+1}>d_{k+k_{4}}$ and $V_{k+k_{4}+1}=RV_{k+k_{4}}$. The angle error $\left|\theta_{k+k_{4}+1}\right|=\left|\vartheta \left(k+k_{4}+1\right)\right|\leq \arcsin\left(\frac{3VT}{2d_{k+k_{4}+1}}\right)\leq \Theta_{k+k_{4}+1,0}$.

**Figure 7:**
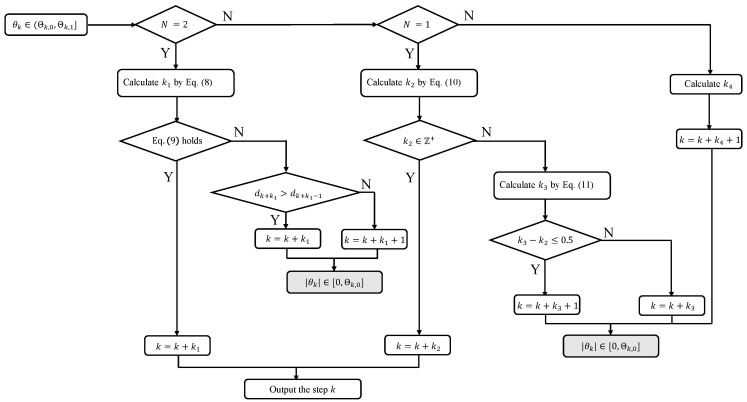
The flowchart of the process for calculating the step *k* in the case of $\theta_{k}\in \left(\Theta_{k,0},\Theta_{k,1}\right]$.

The remaining analyses for N∈{0,1,2} are similar to Section 3.1 where $\left|\theta_{k}\right|\leq \Theta_{k,0}$ and thus are omitted here. Thus, under Assumptions 1 and 2, if $\theta_{k}\in \left(\Theta_{k,0},\Theta_{k,1}\right]$, the TAC problem is solved by controller (3). In particular, if Equation (9) or Equation (10) is satisfied, the TAC problem is solved with the vehicle’s velocity unchanging. Otherwise, the TAC problem is solved with the vehicle rotating the velocity only once. According to the analyses mentioned above, a flowchart is summarized as Figure 7 to show the process for calculating the step *k* in this case. The flowchart of the remaining cases is similar and thus is omitted.

### 3.3. In the Case of $\theta_{k}\in \left(\Theta_{k,1},\pi \right]$

**Figure 8:**
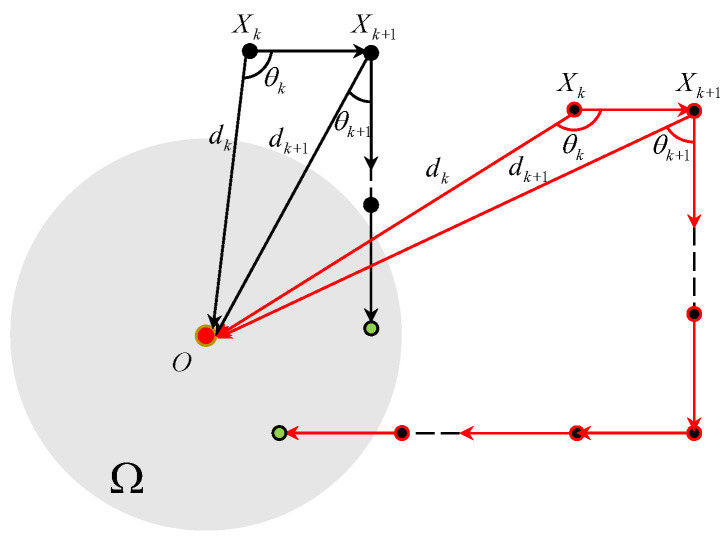
The TAC process for $\theta_{k}\in \left(\Theta_{k,1},\pi \right]$.

Figure 8 plots the TAC process for $\theta_{k}\in \left(\Theta_{k,1},\pi \right]$. In this case, $V_{k+1}=RV_{k}$, $d_{k+1}>d_{k}$ and

(12) $$d_{k+1}=\sqrt{{\left(d_{k}\sin\theta_{k}\right)}^{2}+{\left(VT-d_{k}\cos\theta_{k}\right)}^{2}}$$

holds. It is observed that the trajectory of the TAC process depends on the initial states of the vehicle. Specifically, if

(13) $$d_{k}\cos\theta_{k}\geq VT-\sqrt{\delta^{2}-{\left(VT\right)}^{2}/4},$$

the angle error $\theta_{k+1}=\vartheta \left(k+1\right)\leq \Theta_{k+1,0}$, which coincides with the case where $|\theta_{k}|\;\in \left[0,\Theta_{k,0}\right]$. If

(14) $$\begin{array}{cc} d_{k}\cos\theta_{k} & <VT-\sqrt{\delta^{2}-{\left(VT\right)}^{2}/4},\\ 2d_{k}^{2}\sin\theta_{k} & \geq VTd_{k+1}, \end{array}$$

we have that

(15) $$\begin{array}{cc} \theta_{k+1} & =\vartheta \left(k+1\right)>\Theta_{k+1,0},\\ \theta_{k+1} & =\arccos\left(\frac{d_{k}\sin\theta_{k}}{d_{k+1}}\right)\leq \Theta_{k+1,1}. \end{array}$$

Thus, $\theta_{k+1}\in \left(\Theta_{k+1,0},\Theta_{k+1,1}\right]$ coincides with Section 3.2. Finally, if

(16) $$\begin{array}{cc} 2d_{k}^{2}\sin\theta_{k} & <VTd_{k+1}, \end{array}$$

we have that $\theta_{k+1}\in \left(\Theta_{k+1,1},\pi /2\right]$. Then $V_{k+2}=RV_{k+1}$, $d_{k+2}>d_{k}$ and

(17) $$0<\theta_{k+2}=\vartheta \left(k+2\right)<\Theta_{k+2,0},$$

which coincides with the case where $|\theta_{k}|\;\in \left[0,\Theta_{k,0}\right]$. Thus, under Assumptions 1 and 2, if $\theta_{k}\in \left(\Theta_{k,1},\pi \right]$, the TAC problem is solved by controller (3) with the vehicle rotating the velocity once or twice.

### 3.4. In the Case of $\theta_{k}\in \left(-\pi ,-\Theta_{k,1}\right)$

**Figure 9:**
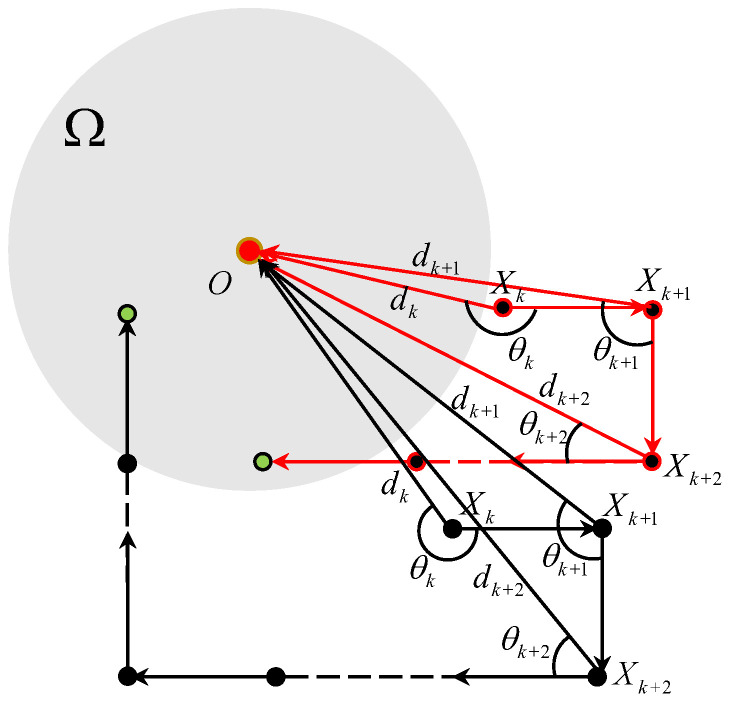
The TAC process for $\theta_{k}\in \left(-\pi ,-\Theta_{k,1}\right)$.

Figure 9 plots the TAC process for $\theta_{k}\in \left(-\pi ,-\Theta_{k,1}\right)$. In this case, $V_{k+1}=RV_{k}$, $d_{k+1}>d_{k}$ and $\theta_{k+1}=\frac{\pi }{2}+\arccos\frac{VT-d_{k}\cos\theta_{k}}{d_{k+1}}$. If $\theta_{k}\in \left(-\pi ,-\Theta_{k,1}\right)$, we have $\theta_{k+1}\in \left(\frac{\pi }{2},\frac{\pi }{2}+\Theta_{k+1,1}\right)\subseteq \left(\Theta_{k+1,1},\pi \right]$. Thus, one obtains that $V_{k+2}=RV_{k+1}$ and $d_{k+2}>d_{k+1}$. The $\theta_{k+2}$ equals to

(18) $$\theta_{k+2}=\arcsin\left(\frac{VT-d_{k}\sin\theta_{k}}{d_{k+2}}\right).$$

If the initial states of the vehicle satisfy

(19) $$d_{k}\sin\theta_{k}\geq VT-\sqrt{\delta^{2}-{\left(VT\right)}^{2}/4},$$

we have that $\theta_{k+2}\in \left(0,\Theta_{k+2,0}\right]$. The corresponding trajectory of the vehicle is depicted by the red solid line of Figure 9. In this case, the TAC problem is solved by the vehicle rotating the velocity twice.

If Equation (19) does not hold, we have $\theta_{k+2}>\Theta_{k+2,0}$. On the other hand, we have

(20) $$\begin{array}{cc} \theta_{k+2} & =\arccos\left(\frac{VT-d_{k}\cos\theta_{k}}{d_{k+2}}\right)\\ \; & \leq \arccos\left(\frac{VT}{2d_{k+2}}\right)=\Theta_{k+2,1}. \end{array}$$

It, together with the fact that $\theta_{k+2}>\Theta_{k+2,0}$, implies that $\theta_{k,2}\in \left(\Theta_{k+2,0},\Theta_{k+2,1}\right]$ which coincides with Section 3.2. The corresponding trajectory of the vehicle is depicted by the black solid line of Figure 9. In this case, the TAC problem is solved with the vehicle rotating the velocity twice or three times.

Thus, under Assumptions 1 and 2, if $\theta_{k}\in \left(-\pi ,-\Theta_{k,1}\right)$, the TAC problem is solved by controller (3) with the vehicle rotating the velocity twice or three times.

### 3.5. In the Case of $\theta_{k}\in \left[-\Theta_{k,1},-\Theta_{k,0}\right)$

**Figure 10:**
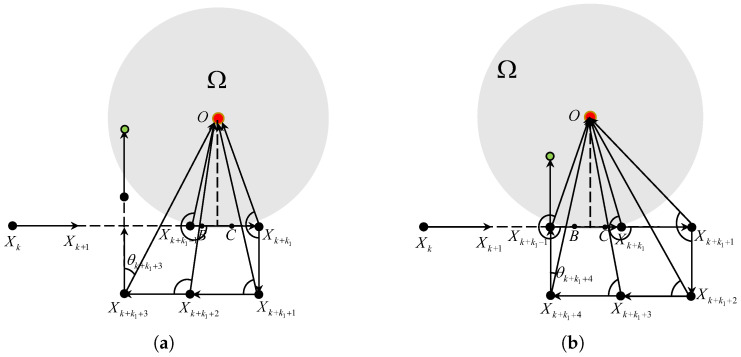
The TAC process for $\theta_{k}\in \left[-\Theta_{k,1},\Theta_{k,0}\right)$ and N=2. (**a**) $\theta_{k+k_{1}-1}<-\Theta_{k+k_{1}-1,1}$. (**b**) $\theta_{k+k_{1}-1}\geq -\Theta_{k+k_{1}-1,1}$.

Similar to Section 3.2, if $\theta_{k}\in \left[-\Theta_{k,1},-\Theta_{k,0}\right)$, the number *N* of the intersection points between the ray $X_{k}X_{k+1}$ and the boundary of Ω satisfies that N∈{0,1,2}. For N=2, similar to Section 3.2.1, if Equation (9) holds, so does $X_{k+k_{1}}\in \Omega$. Conversely, if Equation (9) does not hold $X_{k+k_{1}}\notin \Omega$. Regarding the case, if $\theta_{k+k_{1}-1}<-\Theta_{k+k_{1}-1,1}$, we have $d_{k+k_{1}}>d_{k+k_{1}-1}$ and $V_{k+k_{1}}=RV_{k+k_{1}-1}$. As such, $\theta_{k+k_{1}}\in \left(\pi /2,\pi /2+\Theta_{k+k_{1},1}\right)\subseteq \left(\Theta_{k+k_{1},1},\pi \right]$ holds, then we have $d_{k+k_{1}+1}>d_{k+k_{1}}$, $V_{k+k_{1}+1}=RV_{k+k_{1}}$, and $\theta_{k+k_{1}+1}\in \left(\Theta_{k+k_{1}+1,0},\Theta_{k+k_{1}+1,1}\right]$. It is easily induced that $d_{k+k_{1}+2}<d_{k+k_{1}+1}$, $V_{k+k_{1}+2}=V_{k+k_{1}+1}$, $\theta_{k+k_{1}+2}\in \left(\Theta_{k+k_{1}+2,1},\pi \right)$, $d_{k+k_{1}+3}>d_{k+k_{1}+2}$, $V_{k+k_{1}+3}=RV_{k+k_{1}+2}$, $\theta_{k+k_{1}+3}\in \left[0,\Theta_{k+k_{1}+3,0}\right]$, as plotted in Figure 10a.

For the case of $\theta_{k+k_{1}-1}\geq -\Theta_{k+k_{1}-1,1}$, similar analyses are used to prove that $\theta_{k+k_{1}+4}\in \left[0,\Theta_{k+k_{1}+4,0}\right]$, as plotted in Figure 10b.

The detailed analyses for N={0,1} are similar and thus are omitted here. Thus, under Assumptions 1 and 2, if $\theta_{k}\in \left[-\Theta_{k,1},-\Theta_{k,0}\right)$, the TAC problem is solved by controller (3) with the vehicle rotating the velocity once or three times.

Based on the analysis of Section 3.1, Section 3.2, Section 3.3, Section 3.4 and Section 3.5, it can be concluded that the direction of the vehicle’s velocity is unchanged under the best-case scenario or is adjusted three times under the worst-case scenario during the TAC task. This ends the proof.

**Remark** **3.** *Many target-approach or target tracking controllers in [11,12,13,14,15,16,17] rely on position measurements, which are often unavailable in GPS-denied environments. While our proposed controllers only need the range measurements. The controllers proposed in [25,26] incorporate the sign function, inevitably inducing chattering effects. Moreover, these controllers necessitate exact knowledge of the range information’s time derivative. In contrast, neither the sign function nor the derivative of range measurements is needed in our proposed controller. Some range-only methods in [32,33,34] depend on complex sensor fusion or optimization-based techniques, increasing the computational overhead and reducing real-time applicability. Our proposed controller is computationally lightweight, making it suitable for real-time implementation on resource-constrained platforms.*

**Remark** **4.** *From a practical perspective, the target approach control performance will be affected by measurement noise in ranging by using the proposed controller. As shown in Equation (3), the proposed controller depends on the range measurements between the target and the vehicle at two consecutive sampling times. If there is measurement noise with the maximum value being $d_{max}$, then the proposed controller should be revised as*

(21) $$V_{k}=\left\{\begin{array}{cc} 0 & \;if\;d_{k}\leq \delta -d_{max}\\ V_{k-1} & \;if\;\delta +d_{max}<d_{k}\leq d_{k-1}-2d_{max}\\ RV_{k-1} & \;if\;d_{k}>d_{k-1}+2d_{max} \end{array}\right.$$

*to address the effect of the measurement noise in ranging. A simple explanation for the above controller is as follows. Define $d_{k}$ as $d_{k}=d_{r,k}+d_{n,k}$, where $d_{r,k}$ is the real range and $d_{n,k}$ is the measurement noise with $|d_{n,k}|\;\leq \;d_{max}$. Thus, we have the following results*

(22) $$\begin{array}{cc} d_{k}\leq \delta -d_{max} & \Rightarrow d_{r,k}\leq \delta ,\\ \delta +d_{max}<d_{k} & \Rightarrow \delta <d_{r,k},\\ d_{k}\leq d_{k-1}-2d_{max} & \Rightarrow d_{r,k}\leq d_{r,k-1},\\ d_{k}>d_{k-1}+2d_{max} & \Rightarrow d_{r,k}>d_{r,k-1}. \end{array}$$

*Then, controller (21) is equivalent to*

(23) $$V_{k}=\left\{\begin{array}{cc} 0 & \;if\;d_{r,k}\leq \delta \\ V_{k-1} & \;if\;\delta <d_{r,k}\leq d_{r,k-1}\\ RV_{k-1} & \;if\;d_{r,k}>d_{r,k-1} \end{array}\right.$$

*which has the same form as Equation (3).*

*It is worth noting that due to the existence of the measuring noise, there are two types of uncertain cases during the target approach control.*

*(1) Case 1: When $d_{k}\in \left(\delta -d_{max},\delta +d_{max}\right]$, it is uncertain whether $d_{r,k}\leq \delta$ or not. That means that we are unable to determine whether the vehicle enters the target area.*

*(2) Case 2: When $d_{k}\in \left(d_{k-1}-2d_{max},d_{k-1}+2d_{max}\right]$, it is uncertain whether $d_{r,k}>d_{r,k-1}$ or not. That means that we cannot determine if the vehicle at kT time instant is moving closer to or away from the target relative to (k−1)T time instant.*

*The above two cases demonstrate the limitations of the proposed controller when handling ranging measurement noises, which deserve further investigation in the future.*

## 4. Simulation Results

In this section, several simulation results are presented to validate the effectiveness of the proposed controller (3), along with a comparative analysis against the target tracking algorithm proposed in [35].

**Figure 11:**
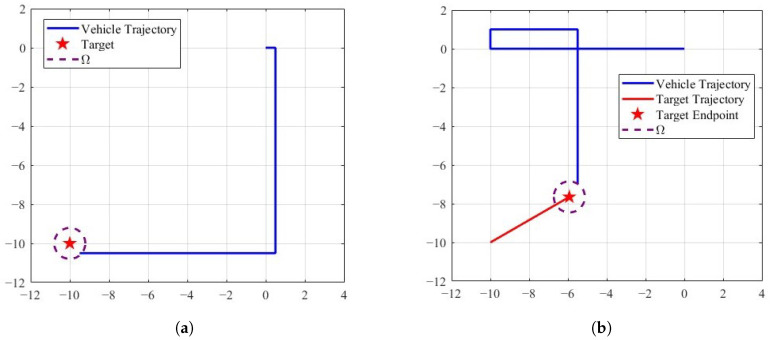
The trajectories of the vehicle for tracking the target under controller (3). (**a**) Static target,. (**b**) moving target.

Figure 11a shows the trajectory generated by the TAC for a static target, where the parameters are set as V=0.5m/s, T=1s, and δ=0.8m. It can be observed that the vehicle reaches the vicinity of the target, requiring only a single directional adjustment. Furthermore, the controller (3) is also applicable in scenarios involving moving targets. As illustrated in Figure 11b, the vehicle, with a velocity of 0.5m/s, successfully approaches the moving target, where the target moves at a velocity of 0.1m/s.

**Figure 12:**
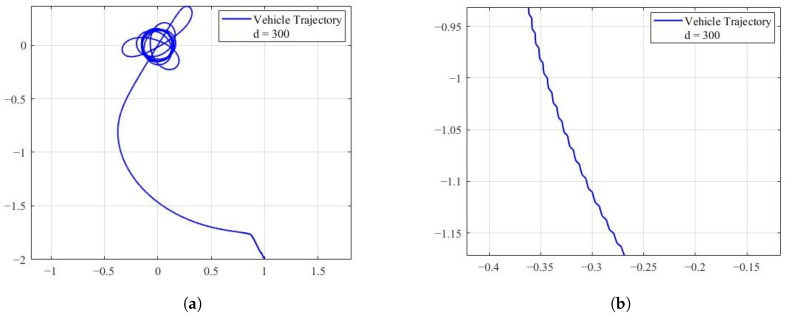
Nonholonomic source seeking with tuning of angular velocity, (**a**) is the parameter used for the simulation: $V_{c}=0.1$, c=100, a=0.5ω=40, R=0.1, h=1, $f^{*}=0$, $q_{r}=1$, $\phi \left(0\right)=26.57^{\circ }$. The symbol $\phi_{0}$ represents the vehicle’s initial angle. (**b**) shows a locally enlarged image of the vehicle’s trajectory.

To provide a comparison, we reproduce the target tracking algorithm proposed in [35], which addresses the problem of guiding a nonholonomic unicycle-type vehicle to track a moving target. In this method, the vehicle maintains a constant forward velocity while adjusting its heading according to the tracking controller. Figure 12 presents the corresponding simulation result. The detailed parameter settings can be found in [35]. As shown in Figure 12, the vehicle successfully converges to the vicinity of the target.

It is worth noting that the controller proposed in [35] has two major limitations. On the one hand, the use of the target tracking algorithm causes the vehicle’s heading to vary sinusoidally, as shown in Figure 12b, resulting in frequent adjustments during the tracking process. On the other hand, the algorithm requires the vehicle’s initial position to be sufficiently close to the target, and its initial orientation to be orthogonal to the direction of the target.

**Figure 13:**
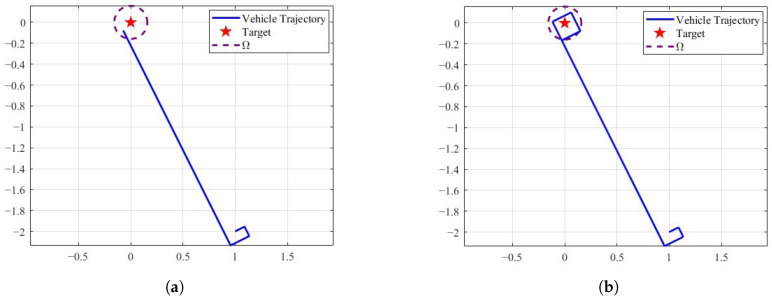
Target approaching control algorithm that relies only on ranging data. (**a**) shows that the vehicle stops moving after entering the target area. The main parameters used are as follows: the vehicle velocity is 0.1 m/s, the sampling time is 1s and $\theta \left(0\right)=26.57^{\circ }$. (**b**) shows that after entering the target area, the vehicle will not be stationary and is able to continuously track the target.

In light of these limitations, we conducted comparative simulations using the same initial conditions for the proposed controller (3). As shown in Figure 13, the TAC algorithm achieves target tracking and exhibits two distinct features: (1) the vehicle maintains straight-line motion between directional changes, and (2) each directional change involves a fixed $90^{\circ }$ rotation.

Utilizing controller (3) ensures that the vehicle maintains a forward trajectory and adjusts its direction to track the target. After entering the target’s area Ω, the vehicle remains stationary, as shown in Figure 13a. If the constraint in Controller (3) that forces the vehicle to remain stationary after entering the area Ω (i.e., when $d_{k}<\delta$, $V_{k}=0$) is removed, the vehicle is able to continuously track the target, as illustrated in Figure 13b.

**Figure 14:**
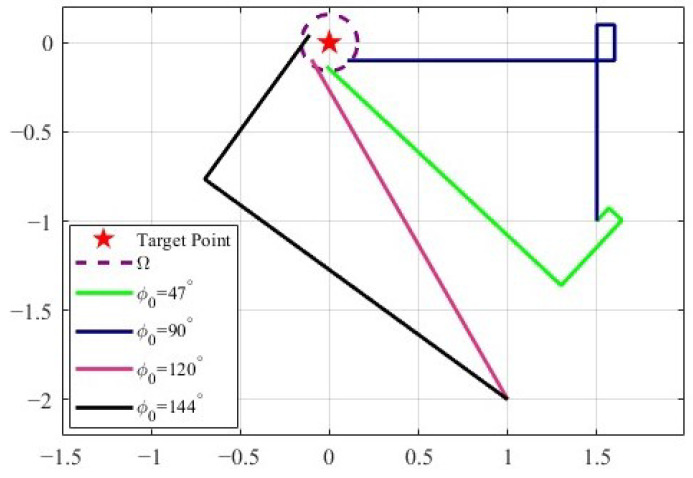
A series of simulations using the TAC algorithm under a range of different initial states. The vehicle velocity is 0.1 m/s. The symbol $\phi_{0}$ represents the vehicle’s initial angle.

In order to further demonstrate the advantage of controller (3) independent of the initial states, we use two different initial positions and four different initial directions for the simulations. The results are shown in Figure 14. It is observed that the vehicle converges to the vicinity of the target with different initial states. Thus, the simulation results indicate the proposed controller is independent of the initial states.

## 5. Experiments

**Figure 15:**
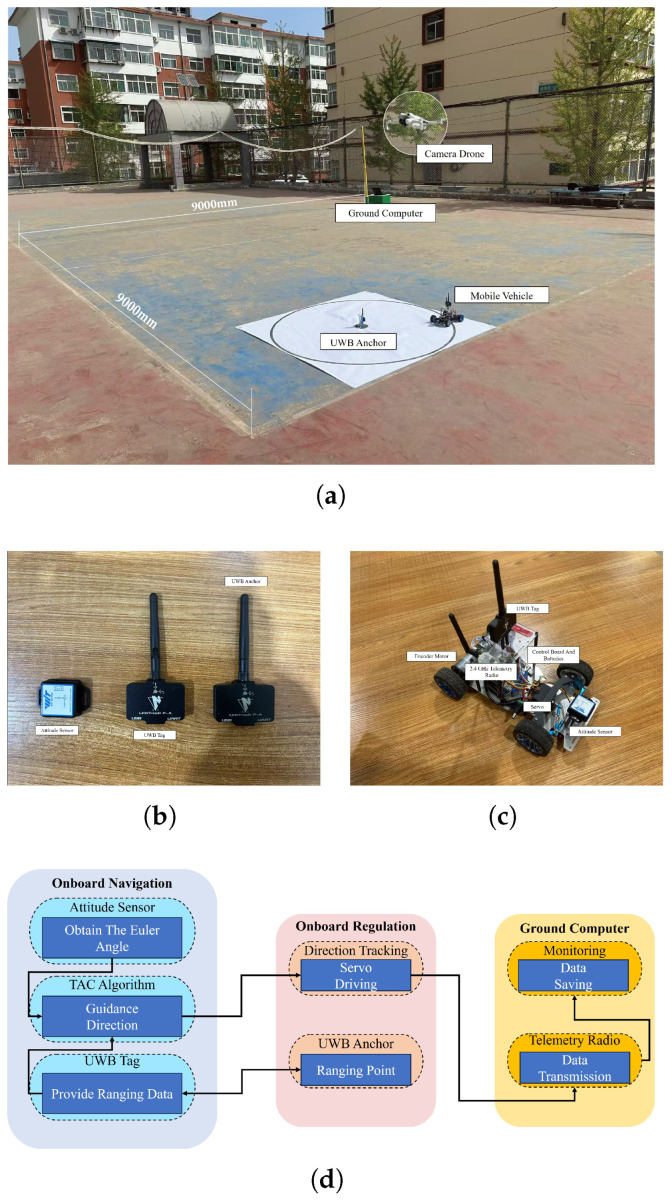
(**a**) The platform consists of a mobile vehicle, a DJI Mini4 Pro UAV, a computer, a circular area with a radius of $\delta =\sqrt{\frac{5}{2}}VT$ is used to mark the visual range of the target point, and a 9 m × 9 m square open space. (**b**) The mobile vehicle is equipped with two kinds of sensors, the ranging sensor based on Ultra Wide Band (UWB) technology is used by the TAC algorithm to determine whether a π/2 degree clockwise rotation is required and the attitude sensor is used to determine whether the task of rotating a π/2 degrees has been completed. (**c**) Size: 290 mm (length) × 195 mm (width) × 180 mm (height) and detailed components of the mobile vehicle. (**d**) Structure of the target approaching control system.

In this section, we validate the effectiveness of controller (3) by mobile vehicle outdoor experiments. As shown in Figure 15a, the experiment platform is composed of a 9 m × 9 m open space, a mobile vehicle to execute the TAC task by controller (3), a ground computer to receive information from the mobile vehicle, and a camera drone (DJI Mini 4 Pro (Shenzhen, China)) to record the tracking trajectory of the mobile vehicle. For ease of visibility, a circular area with a white background and a black edge is used to represent the target area (2). It is observed in Figure 15b that the mobile vehicle is equipped with two encoder motors (12 V), a 2200 mAh lithium battery, a control module (STM32F1 (STMicroelectronics, Geneva, Switzerland)), an Ackerman steering servo, and a 2.4 GHz telemetry radio (LR-24 (Micoair, Shenzhen, China)). As demonstrated in Figure 15c, the attitude sensor and the ranging sensor based on Ultra Wide Band (UWB) technology are used to provide the bearing and ranging information, respectively. Figure 15d exhibits the structure of the UV target approaching control system, which is divided into three parts: the onboard navigation to produce desired guidance direction based on TAC, the onboard regulation to drive the servo to rotate and launch transmits data, and the ground computer to receive and save data.

In what follows, two typical scenarios are provided to demonstrate the performance of the TAC controller (3). The first scenario corresponds to the cases where $\theta_{k}\in \left(\Theta_{k,0},\Theta_{k,1}\right]$ and N=0, as shown in Figure 6, and the second scenario corresponds to the cases where $\left[-\Theta_{k,1},-\Theta_{k,0}\right)$ and N=0. The experiment parameters are set as V=0.5 m/s, T=1 s, and δ=80 cm. It is easily observed that the condition (4) is fulfilled.

### 5.1. The First Scenario

**Figure 16:**
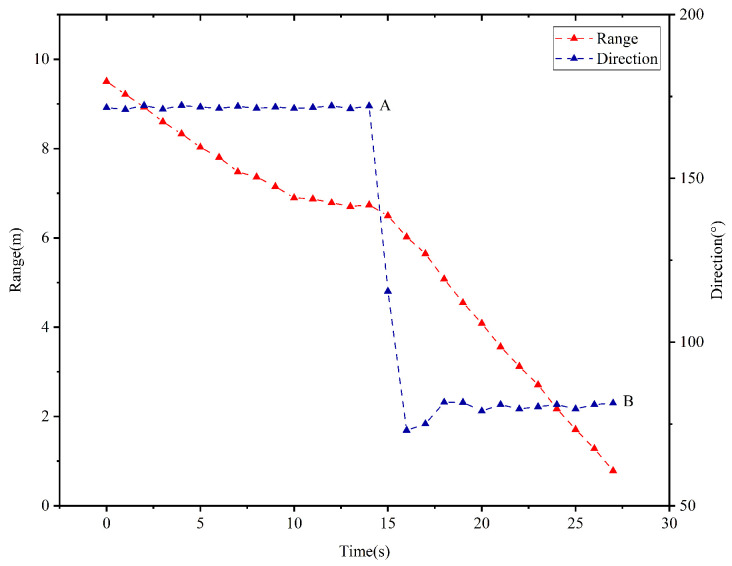
Temporal evolution of the range from the vehicle to the target and the vehicle’s moving direction in the first scenario.

In this case, the vehicle rotates only once by an angle of π/2 in the clockwise direction before the vehicle enters the target area. The temporal evolution of the vehicle’s moving direction and the range from the vehicle to the target is depicted in Figure 16. When kT≤13 s, the range $d_{k}$ between the vehicle and the target decreases as time evolves, and the vehicle’s moving direction remains unchanging. As observed in the point *A* of the Figure 16, the range $d_{14}>d_{13}$ holds when kT=14 s. According to controller (3), the vehicle rotates by an angle of π/2 in the clockwise direction from $174^{\circ }$ to $84^{\circ }$ during the time interval [14,16]. When kT∈[16,27] s, the range $d_{k}$ decreases and the vehicle’s moving direction remains unchanged. As observed in the point *B* of the Figure 16, the range $d_{27}=78.8<80$ cm is held at kT=27 s. As such, the vehicle enters the target area, and the TAC problem is solved.

In this scenario, the vehicle executes a 90-degree clockwise rotation during the 14–16 s interval, which demonstrates mild discrepancies when compared with theoretical analysis in the previous section. This is due to the fact that the mobile vehicle of the experimental platform constitutes a typical under-actuated system, indicating that it lacks the capability to directly execute 90-degree directional modifications. Similar phenomena are manifested in the subsequent experimental scenario.

### 5.2. The Second Scenario

**Figure 17:**
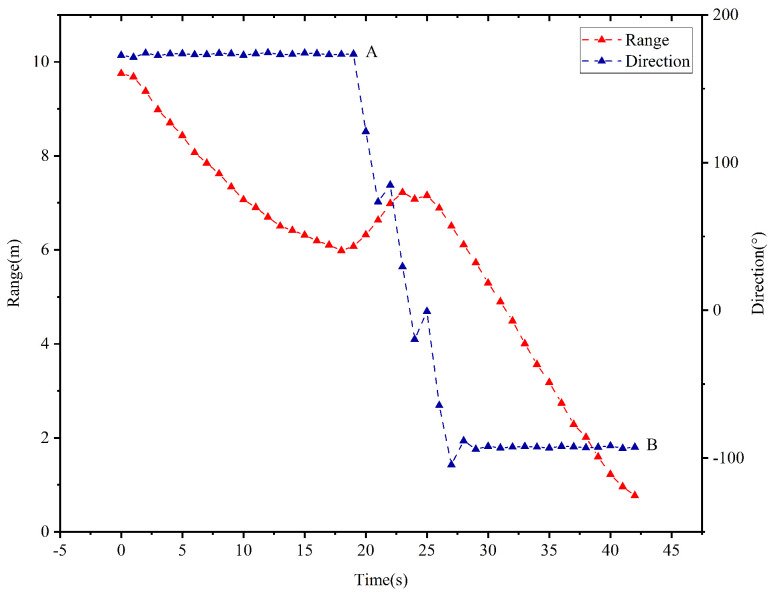
Temporal evolution of the range from the vehicle to the target and the vehicle’s moving direction in the second scenario.

In this case, the vehicle will rotate three times by an angle of π/2 in the clockwise direction before the vehicle enters the target area. The temporal evolution of the vehicle’s moving direction and the range from the vehicle to the target is depicted in Figure 17. When kT≤18 s, the range $d_{k}$ between the vehicle and the target decreases as time evolves, and the vehicle’s moving direction remains unchanging. As observed in the point *A* of the Figure 17, the range $d_{19}>d_{18}$ holds when kT=19 s. According to controller (3), the vehicle rotates by an angle of π/2 in the clockwise direction during the time interval [19,21] s. Similarly, rotation is executed during the time interval [22,24] s and [25,27] s due to $d_{22}>d_{21}$ and $d_{25}>d_{24}$. When kT∈[27,42] s, the range $d_{k}$ decreases and the vehicle’s moving direction remains unchanged. As observed in the point *B* of the Figure 17, the range $d_{42}=77.5<80$ cm is held at kT=42 s. As such, the vehicle enters the target area, and the TAC problem is solved.

## 6. Conclusions

In this paper, we have demonstrated a range-based control strategy for guiding a discrete-time first-order vehicle system toward a static circular target area without relying on absolute bearing or position data. Theoretical analysis confirms that the proposed controller ensures target approaching in finite steps. Specifically, the velocity direction remains unchanged in the best scenarios, while requiring at most three adjustments in the worst case. Finally, the mobile vehicle experiments have corroborated the theoretical findings through two elaborate scenarios. This paper provides an effective solution for target approaching control with limited sensing capabilities, balancing computational simplicity with guaranteed convergence. First-order integrator systems are considered in this paper. It is more practical to extend the results to complicated dynamic systems and environment with the obstacles, which will be studied in the future work.

## Data Availability

The video data of the outdoor mobile vehicle experiment are available at https://www.bilibili.com/video/BV14H7Az2E7r/ (accessed on 31 May 2025). The study data can be obtained upon request from the corresponding author.
